# Influence of Welding Speed on Fracture Toughness of Friction Stir Welded AA2024-T351 Joints

**DOI:** 10.3390/ma14061561

**Published:** 2021-03-22

**Authors:** Miodrag Milčić, Dragan Milčić, Tomaž Vuherer, Ljubica Radović, Igor Radisavljević, Aleksija Đurić

**Affiliations:** 1 Faculty of Mechanical Engineering, University of Nis, Aleksandra Medvedeva 14, 18000 Niš, Serbia; dragan.milcic@masfak.ni.ac.rs; 2 Faculty of Mechanical Engineering, University of Maribor, Smetanova 17, 2000 Maribor, Slovenia; tomaz.vuherer@um.si; 3 Military Technical Institute, Ratka Resanovića 1, 11000 Belgrade, Serbia; ljubica.radovic@vti.vs.rs (L.R.); igor.radisavljevic@vti.vs.rs (I.R.); 4 Faculty of Mechanical Engineering, University of East Sarajevo, Vuka Karadžića 30, 71123 East Sarajevo, Bosnia and Herzegovina; aleksija.djuric@ues.rs.ba

**Keywords:** friction stir welding, process parameters, welding speed, tool rotation speed, AA-2024-T351, fracture toughness

## Abstract

In order to ensure a quality welded joint, and thus safe operation and high reliability of the welded part or structure achieved by friction stir welding, it is necessary to select the optimal welding parameters. The parameters of friction stir welding significantly affect the structure of the welded joint, and thus the mechanical properties of the welded joint. Investigation of the influence of friction stir welding parameters was performed on 6-mm thick plates of aluminum alloy AA2024 T351. The quality of the welded joint is predominantly influenced by the tool rotation speed *n* and the welding speed v. In this research, constant tool rotation speed was adopted *n* = 750 rpm, and the welding speed was varied (*v* = 73, 116 and 150 mm/min). By the visual method and radiographic examination, imperfections of the face and roots of the welded specimens were not found. This paper presents the performed experimental tests of the macro and microstructure of welded joints, followed by tests of micro hardness and fracture behavior of Friction Stir Welded AA2024-T351 joints. It can be concluded that the welding speed of *v* = 116 mm/min is favorable with regard to the fracture behavior of the analysed FSW-joint.

## 1. Introduction

Aluminum is one of the most common metals on earth and is widely used for engineering structures and components in many industries such as aerospace, automotive, rail vehicles, and shipbuilding. Aluminum and aluminum alloys have low density, high corrosion resistance, excellent processing properties and excellent thermal and electrical properties [1]. Aluminum is intrinsically sustainable because once produced, it can be recycled repeatedly without any loss in quality and reused in the manufacturing of consumer and industrial products. The fabrication of light structures from Al alloys is usually done by welding the parts. Various welding processes are used, most often electric arc welding (Metal Inert Gas—MIG and Tungsten Inert Gas—TIG), friction stir welding (FSW), friction joining. Many aluminum alloys, such as the 2XXX and 7XXX series, are known for their low welding capabilities and are often classified as non-weldable materials when electric arc processes are used [2]. The problem of non-weldable aluminum alloys of the 2XXX and 7XXX series has conditioned the research of new welding technologies in the last thirty years. The friction stir welding (FSW) process is a relatively new joining technique [3]. Friction stir welding (FSW) is a welding process developed and patented by The Welding Institute (TWI) in the United Kingdom in December 1991. This welding process was developed by Wayne M. Thomas with his team of researchers and engineers. The main metallurgical advantage of this new process is that the welding is performed in the solid state, not reaching the melting point of the base material, which leads to less distortion, less residual stresses and less welding defects compared to other fusion welding techniques [4,5]. For this reason, this relatively new procedure is very well accepted in the industry, especially in the aerospace sector which still uses rivets and bolts in many structural components [6]. Extensive research is being conducted in this area to study the applicability of this welding process in industrial applications [7]. Materials that were once considered non-weldable with fusion welding processes, now can be welded using the friction stir welding process [8,9,10,11].

The input parameters required to produce a welded joint by the FSW welding process are tool rotation speed, translational tool speed or welding speed, axial force, immersion depth, tool angle, geometry and characteristics of the tool shoulder and tool pin [12]. Numerous studies have been performed in the field of the influence of FSW welding parameters on the mechanical properties and microstructure of the welded joint [13,14,15,16,17,18]. An optimal ratio between tool rotation speed and welding speed *n*/*v* is required to guarantee good material mixing and heat input along the entire length of the welded joint [19]. This *n*/*v* (weld pitch) ratio is directly proportional to the heat generated in the welded joint [20]. According to Vilaca et al. [21], FSW welding conditions are classified depending on the *n*/*v* ratio ranging from hot to cold welds. Kosturek et al., in [22], investigate the influence of friction stir welding parameters on the microstructure and mechanical properties of alloy AA2519 modified with 0.16% Sc. Welding conditions were in the domain of non-hot welding with tool rotation speeds of 400, 600, 800, 1000, 1250, and 1500 rpm. In the paper [23], Khodir et al. provide research on the influence of tool rotation speed (400, 600, 800, 1000, 1250, and 1500 rpm), at a constant welding speed of 50 mm/min on the microstructure, hardness distribution and tensile properties of FSW butt-welded plates with a thickness of 3-mm alloy AA2024-T3. In the paper [24], Perović et al. give the influence of welding speed on the mechanical properties of the welded joint achieved by FSW of Al–Zn–Mg–Cu alloy (EN AW 7049). Tests of the influence of welding parameters (constant tool rotation speed and different welding speed) on tensile properties, hardness profile, impact toughness and fracture mechanics parameters were performed. In the paper [25] Barenji gives the results of research on the influence of the speed of translational movement of tools—the speed of friction stir welding on the microstructure and mechanical properties of aluminum alloy 7020-T6. For this purpose, 5-mm thick plates were welded at welding speeds of 50, 100, 150 and 200 mm/min, and the tool rotation speed was kept constant at 900 rpm. In the paper [26], investigations of the influence of tool rotation speed and welding speed on the microstructural and mechanical properties of butt joints of 1.5-mm thick sheet metal made of 2024-T4 aluminum alloy are given. Five tool rotation speeds ranging, from 560 to 1800 rpm, and five welding speeds, from 11 to 45 mm/min, were used. Cao and Jahaziin [27], investigate the effect of welding speed ranging from 5 to 30 mm/s on 2-mm butt joint quality of friction stir welded AZ31B-H24 magnesium alloy was investigated to determine defects, microstructures, hardness and tensile properties.

In addition, researching the mechanical properties of FSW welded joints, knowledge of fatigue load behavior is essential for aircraft structures. Numerous studies have been performed in the last decade to study the high-cycle fatigue of FSW welded joints made of aluminum alloys 2XXX [28,29,30], with special emphasis on the correlation between the microstructure of the material with the initiation and growth of the fatigue crack [31,32,33]. Aydin et al. [34] investigated the influence of welding parameters on the fatigue behavior of FSW-AA2014-T6 alloy, and Moghadam et al. [35] investigated the influence of welding parameters on fracture toughness and fatigue crack growth of FSW-AA2024-T351 alloy. José et al. [36] investigated characterizing the fatigue crack growth behavior of the 6082-T6 aluminum alloy. Zhiqiang et al. [37] investigates the effects of axial ultrasonic vibration on the microstructure evolution, residual stresses distribution and fatigue fracture behavior of a 7N01-T4 joint.

Some aspects of FSW-AA2024-T351-joints have already been investigated by the authors of this paper in the past. Milčić et al. [38,39] have investigated the influence of the welding speed on the material microstructure, hardness distribution and Charpy impact toughness of the analyzed FSW-joint. In the subsequent studies [40,41], Milčić et al.l investigated the fatigue behavior (determination of S-*n* curves) of the analyzed FSW-joint. Based on these experimental results, the highest fatigue strength was found for the welding speed of 116 mm/min. The effect of welding speed ranging from 73 to 150 mm/min on the butt joint quality is studied in terms of microstructure, hardness and fracture toughness properties.

## 2. Material and Experimental Procedure

### 2.1. Material

The chemical compositions and mechanical properties of these base materials are given in Table 1 and Table 2.

The dimensions of welded plates were 500 mm × 65 mm × 6 mm. Both sides of the welding plates were machined on the grinder at a thickness of 6 mm.

### 2.2. Friction Stir Welding

**Figure 1:**
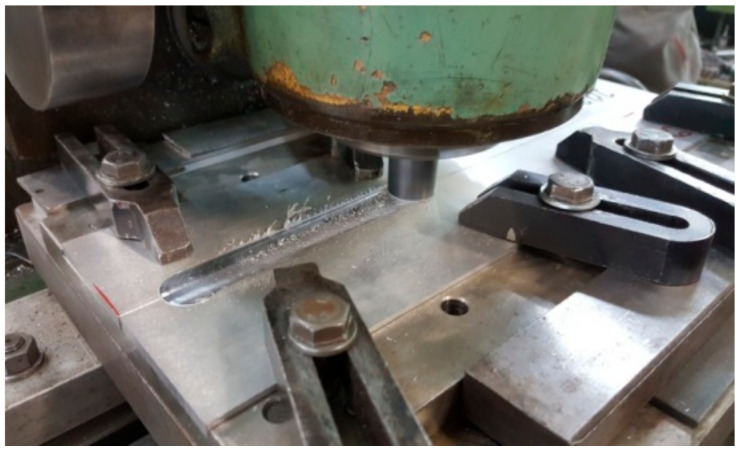
Conventional Milling Machine for friction stir welding (FSW).

**Figure 2:**
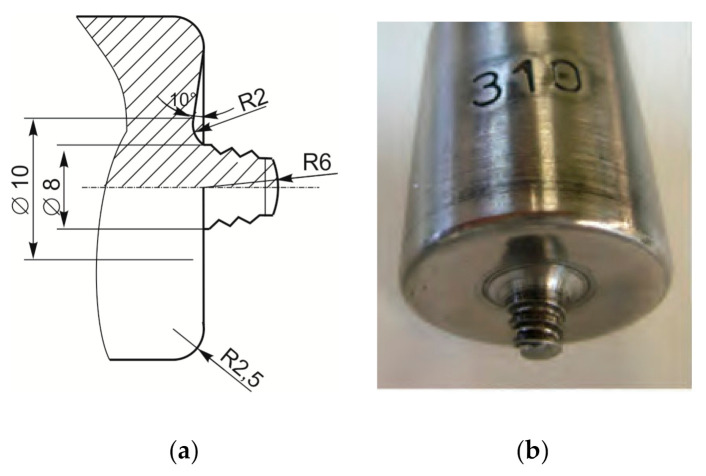
FSW tool geometry (**a**); FSW tool (**b**).

Before the start of welding, an austenitic plate is placed under the welding plates as a backing plate. A milling machine was used for welding. The weld length was approximately 400 mm. Figure 1 shows a conventional milling machine (ALG 200, Prvomajska, Croatia) and Figure 2 a tool used for the butt joint FSW. The tool is made of 55CrMo8 steel and heat-treated to hardness of 50 HRc. The tool has a conical pin and standard thread.

The experiment was aimed to find the influence of input kinematic parameters such as welding speed (*v*) and tool rotation speed (*n*) on metallurgical and mechanical characteristics of welded joints. Welding was performed with a constant tool rotation speed (*n* = 750 rpm) and with variations in the welding speed (*v* = 73, 116 and 150 mm/min), which is showed in Table 3. The other parameters of welding were maintained constantly.

### 2.3. Microstructural Analysis

After the welding process was completed, welds were tested. All welds were examined using visual control and X-ray radiographs before machining into specimens and no any apparent defects were found.

Microstructural observation was carried out on the cross-section obtained by transversely cutting the joint. Sanding was performed on sandpaper of fineness P150, P240, P320, P400, P600, R1200 and R2500. Diamond pastes with a granulation of 7/5 μm and 5/3 μm for final polishing were used for polishing the sample. In order to obtain a better surface after polishing with diamond paste, the technique of electrochemical polishing and etching was applied. Electrochemical polishing and etching were performed on a Struers Polectrol device (Struers, København, Danmark). An aqueous solution of perchloric acid was used for electrochemical polishing, and Barker’s reagent (5 mL HBF_4_, 200 mL H_2_O) was used for etching. The microstructural observation was conducted by using an optical microscope Epiphot-300 (Nikon, Tokyo, Japan).

### 2.4. Hardness Testing

**Figure 3:**
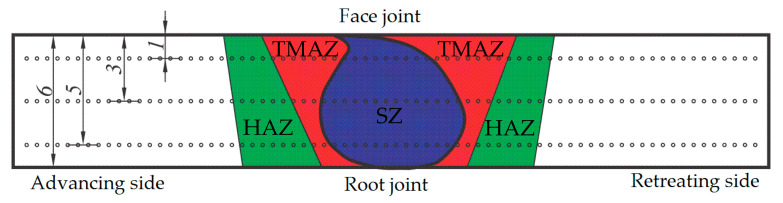
Microhardness measurement scheme with characteristic zones of welded joint (SZ—stirred zone, TMAZ—thermomechanical affected zone, HAZ—heat affected zone).

Hardness testing was carried out on the cross-section obtained by transversely cutting the joint. The hardness tests were conducted by using a HVS-1000 micro Vickers hardness tester (TIME, Beijing, China) with a load of 9807 N for 15 s. The hardness profile was examined along three horizontal directions: the weld face (1 mm from the face of the welded joint), the weld center (3 mm from the face of the welded joint) and the weld root (5 mm from the face of the welded joint) (Figure 3). The distance between the prints was 0.5 mm.

### 2.5. Fracture Testing

**Figure 4:**
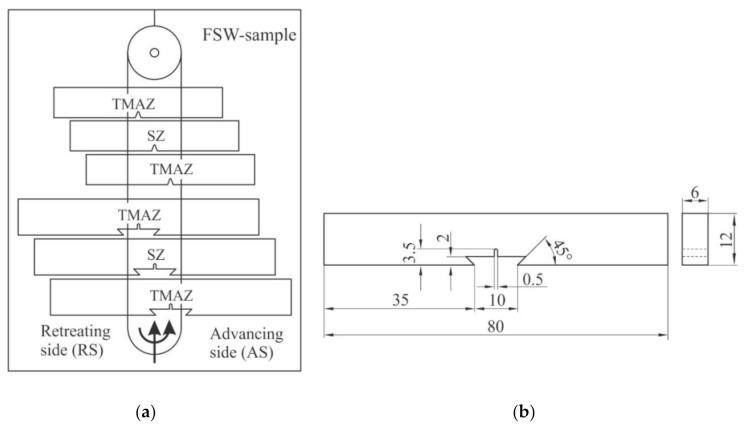
Subtraction of the plate specimen (**a**); specimen for the fracture toughness testing (**b**). (TMAZ—notch position 4 mm from center of weld on advancing side (AS) and retreating side (RS); SZ—notch position in center of weld).

The effect of heterogeneity of the structure and mechanical properties of the welded joint determines the position of formation, and then the crack propagation. Given the heterogeneous structure of the welded joint, the fracture mechanics parameters were determined in different parts of the welded joint (Figure 4a): the middle of the welded joint, the advancing side (AS) of the welded joint (4 mm from the middle of the welded joint) and the retreating side (RS) of the welded joint (4 mm from the middle of the welded joint).

Fracture toughness of the FSW-joint was evaluated on specimens according to the ASTM E1820 Standard [43], shown in Figure 4b, with a thickness of 6 mm. An initial notch (3.5 mm long and 0.5 mm wide) was produced using the Wire Electrical Discharge Machining (WEDM)-technology and served as a seam to produce a fatigue crack in the range 5.4–6.6 mm on a Rumul Cracktronic device (Russenberger Prüfmaschinen AG, Neuhausen am Rheinfall, Switzerland) under pulsating loading (loading ratio R = 0.1).

**Figure 5:**
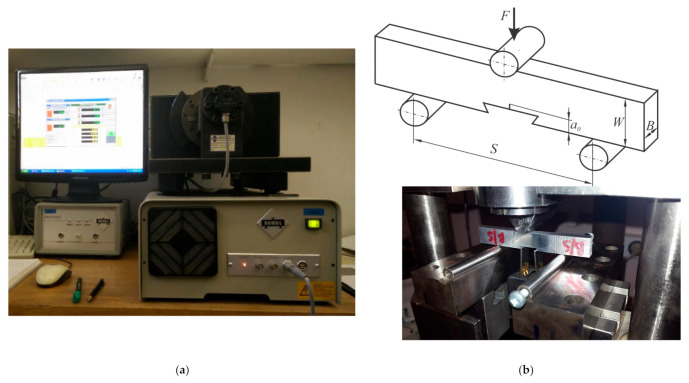
(**a**) Fracture toughness testing setup on a Rumul Cracktronic; (**b**) bend test fixture design. (Specimen: *B*—width; *W*—height; *a*_0_—initial crack size; *S*—support distance; *F*—load).

Fracture toughness testing of the FSW samples was carried out using a Rumul Cracktronic, as shown in Figure 5a and according to the ASTM standard E1820-20 [43].

The range of the initial fatigue crack length corresponded to the guidelines reported in the ASTM E1820 Standard [43], where the following condition should be satisfied: 0.45 ≤ *a*_0_/*W* ≤ 0.55 (*W* =12 mm for the specimen in Figure 4b). Prepared specimens were then subjected to the bending loading at room temperature on round supports *S* = 4·*W* = 4·12 = 48 mm (Figure 5b) apart with the load applied in the middle, on the opposite side of the initial crack. Bending force and Crack Mouth Opening Displacement (CMOD) were measured until breakage as specified in the ASTM E1820 Standard [43] (Figure 5b).

## 3. Results and Discussion

### 3.1. Microstructural Analysis

**Figure 6:**
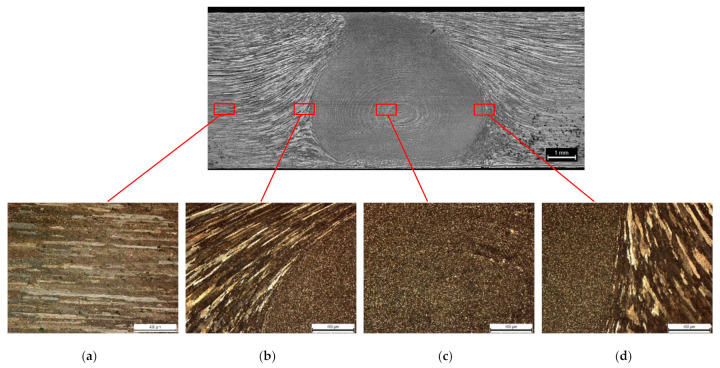
Microstructure of the analyzed FSW-joint at welding speed *v* = 73 mm/min: (**a**) base material (BM); (**b**) thermo-mechanically affected zone (TMAZ)- stir zone (SZ); (**c**) stir zone (SZ); (**d**) stir zone (SZ)—thermo-mechanically affected zone (TMAZ).

The appearance of the microstructure in different areas of the FSW welded joint of alloy 2024-T351 is shown in Figure 6 for the welding sample A. In the structure of the weld a large non-homogeneity can be observed. The grains are of various shapes, from equiaxed, in the nugget (Figure 6c), to very elongated, in the thermomechanical affected zone (TMAZ) (Figure 6b,d). In the base material (BM), because of the previously performed rolling process, the structure consists of elongated (BM) grains (Figure 6a).

### 3.2. Effects Welding Parameters of FSW on Microhardness

**Figure 7:**
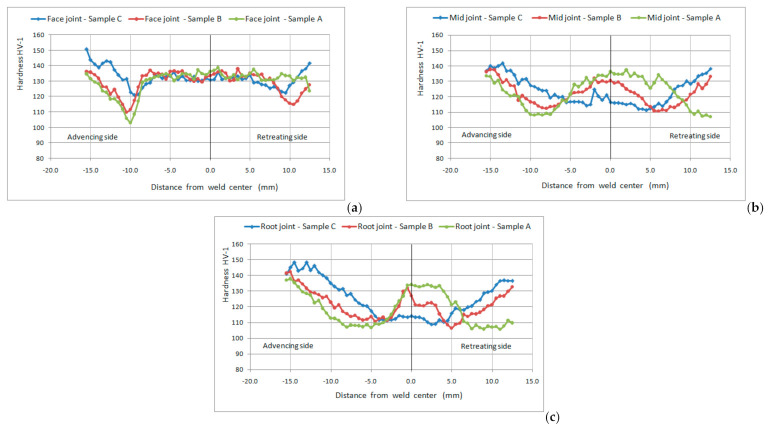
Hardness profile of the welded joint for different welding parameters obtained by measuring at (**a**) a distance of 1 mm from the face of the welded joint; (**b**) a distance of 3 mm from the face of the welded joint; (**c**) a distance of 5 mm from the face of the welded joint.

Results of the measured hardness values of the welded joint, for all three welded samples, are shown in Figure 7.

At a constant tool rotation speed, with the increase in the welding speed, the generated amount of heat during welding decreases. Increasing the welding speed has a great influence on the hardness of the stirred zone (SZ) and the thermomechanical affected zone (TMAZ), but a weak influence on the heat affected zone (HAZ). The hardness level in the SZ and the TMAZ decreases with increasing welding speed (Figure 7a–c). Softened HAZ, on both sides of the middle of the welded joint, increases slightly with increasing welding speed (with decreasing heat input) [38,39].

For a constant tool rotation speed, the highest amount of heat is generated at the lowest welding speed. For this case, the grain size in the SZ is the largest. It is expected that the hardness decreases due to the coarser grain. However, the hardness increases. This tendency suggests that a significant increase in hardness with decreasing welding speed is not a function of grain size, but a function of the size and distribution of second phase particles and precipitates. Similar conclusions can be seen in the paper [23], Khodir et al.

In Figure 7a, in which the hardness profile was obtained by measuring at a distance of 1 mm from the face of the welded joint, it can be seen that in the area below the tool shoulder (TMAZ and SZ) for all welding parameters the hardness range is from 130 HV to 135 HV. The hardness in the SZ is highly dependent on the welding speed (Figure 5). In the case of the lowest welding speed (highest heat input), the level of hardness in the SZ is the highest. This can be clearly seen in the hardness profile obtained by measuring in the middle of the welded joint and in the root of the welded joint (Figure 7b,c).

### 3.3. Effects Welding Parameters of FSW on Fracture Toughness

**Figure 8:**
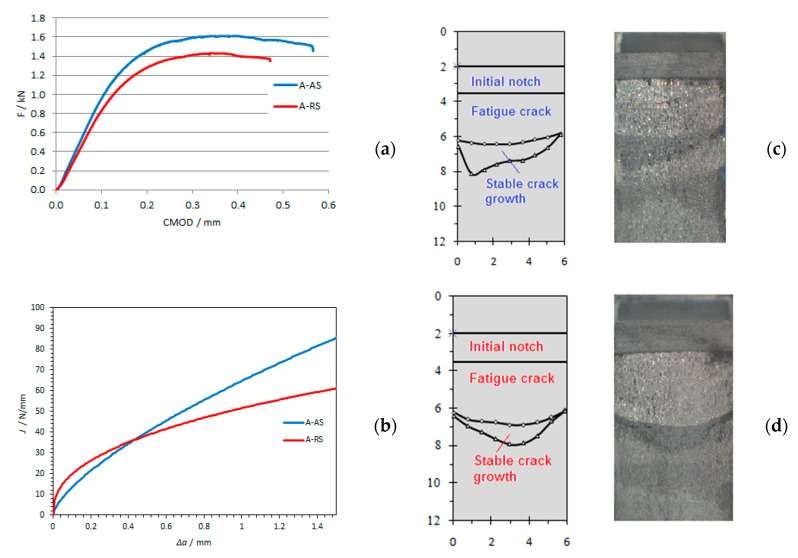
(**a**) Experimental data of load-displacement for specimens for welding speed *v* = 73 mm/min; (**b**) Comparisons of J-R curves for specimen A-AS and A-RS; (**c**) crack surfaces on advancing side (AS)for specimen A-AS; (**d**) crack surfaces on advancing side (AS)for specimen A-RS. (A-AS—specimen for welding speed 73 mm/min (A)—notch position 4 mm from center of weld on advancing side (AS); A-RS—welding speed 73 mm/min (A)—notch position 4 mm from center of weld on retreating side (RS)).

**Figure 9:**
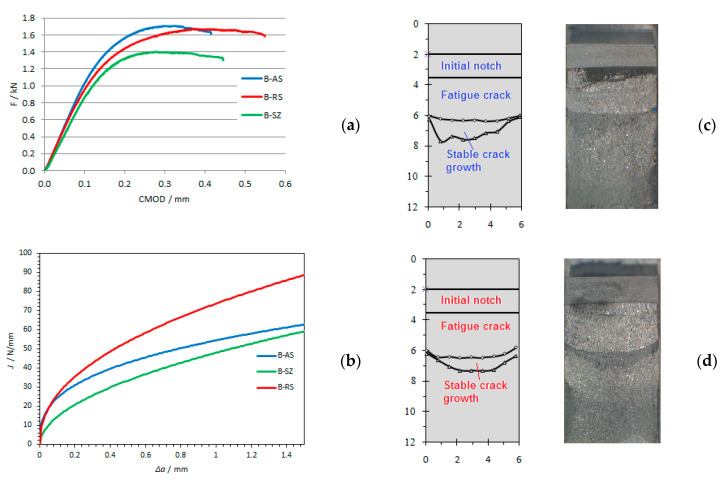
(**a**) Experimental data of load-displacement for specimens for welding speed *v* = 116 mm/min; (**b**) comparisons of J-R curves for specimen B-AS, B-RS and B-SZ; (**c**) crack surfaces on retreating side (RS) for specimen B-AS; (**d**) crack surfaces on retreating side (RS) for specimen B-RS. (B-AS—specimen for welding speed 116 mm/min (B)—notch position 4 mm from center of weld on advancing side (AS); B-RS—welding speed 116 mm/min (B)—notch position 4 mm from center of weld on retreating side (RS); B-SZ—welding speed 116 mm/min (B)—notch position in center of weld (SZ)).

**Figure 10:**
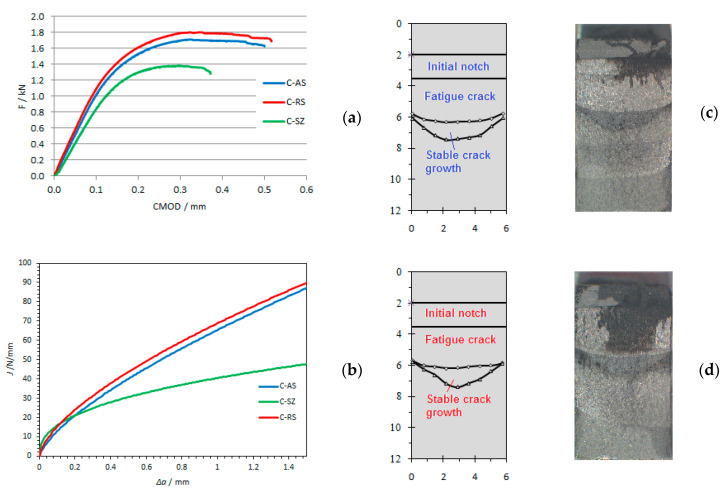
(**a**) Experimental data of load-displacement for specimens for welding speed *v* = 150 mm/min; (**b**) comparisons of J-R curves for specimen C-AS, C-RS and C-SZ; (**c**) crack surfaces on retreating side (RS) for specimen C-AS; (**d**) crack surfaces on retreating side (RS) for specimen C-RS. (C-AS—specimen for welding speed 150 mm/min (C)—notch position 4 mm from center of weld on advancing side (AS); C-RS—welding speed 150 mm/min (C)—notch position 4 mm from center of weld on retreating side (RS); C-SZ—welding speed 150 mm/min (C)—notch position in center of weld (SZ)).

Specimens after testing were then subjected to the temperature 200 °C to oxidize the crack surface, which enabled better visualization of the remaining ligament (Figure 8, Figure 9 and Figure 10).

Obtaining critical values of *J*-integral, *J_IC_*, as well as critical values of crack opening, *δ_Ic_* for different structures of welded joints was obtained at different welding speeds. The critical values of the *J*-integral—*J_Ic_* are determined based on the ASTM E1820 standard and are shown in Table 4. Fracture toughness *K_Ic_* is determined by the expression:

(1) $$K_{Ic}=\sqrt{\frac{E\cdot J_{Ic}}{1-\nu^{2}}}$$

where: *v*—the Poisson’s ratio; *E*—modulus of elasticity (MPa).

**Figure 11:**
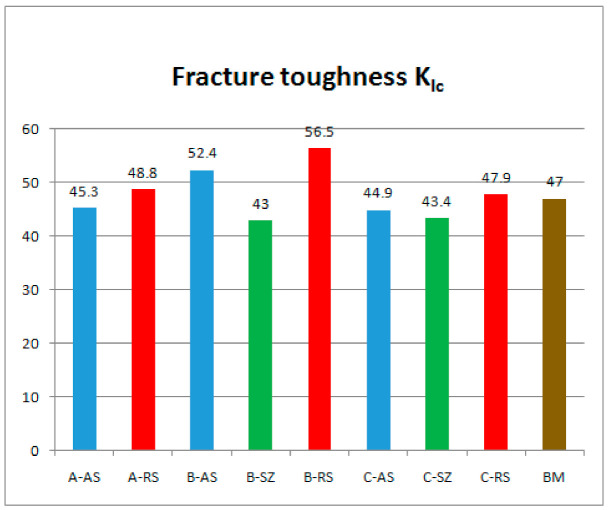
Fracture toughness values K_Ic_ for welded joints achieved by different welding parameters and different notch positions.

By analyzing the results of fracture toughness, it can be concluded that for all welding samples, the structure of the welded joint on the retreating side of the welded joint has the highest fracture toughness (Figure 11), i.e., it has a more pronounced resistance to crack propagation than the structure on the advancing side of welded joint in all tested samples. This is consistent with the obtained fracture toughness values in the work of Moghadam, D.G. et al. [35].

**Figure 12:**
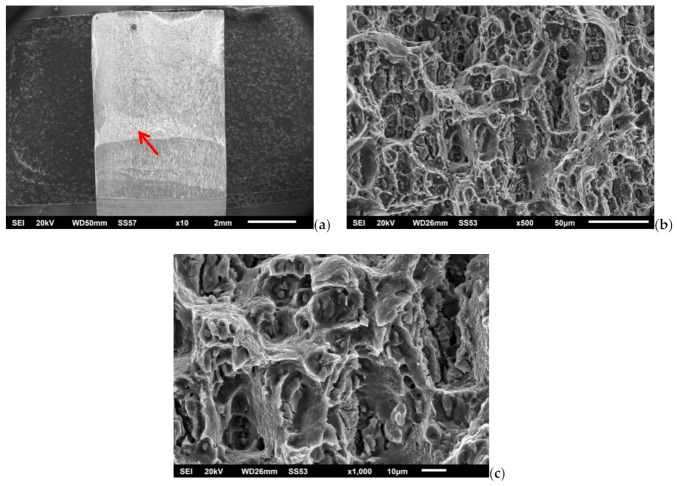
Fracture surface of the B-AS specimen: (**a**) macro, (**b**) and (**c**) stable crack growth zone.

Perović in [24] gives the results of fracture toughness of the FSW joint, with higher values on the retreating side in accordance with the results of this research. Similar fracture toughness results for joint and base metal of FSWed AA2024-T351 were obtained by Moghadam and Farhangdoost [35] for the initial crack in the lump zone and the orientation of the cracks along the welding direction. After the experimental determination of fracture mechanics parameters in the conditions of elastic–plastic fracture mechanics defined by the ASTM E 1820 standard, SEM fractographic analysis of fracture surfaces of Single Edge Notched Bend (SENB) specimens for some structures of FSW welded joint was performed. Figure 12a shows the macroscopic view of the fracture surface of the specimen B-AS, while Figure 12b,c shows the stable crack growth zone of this specimen at higher magnification.

**Figure 13:**
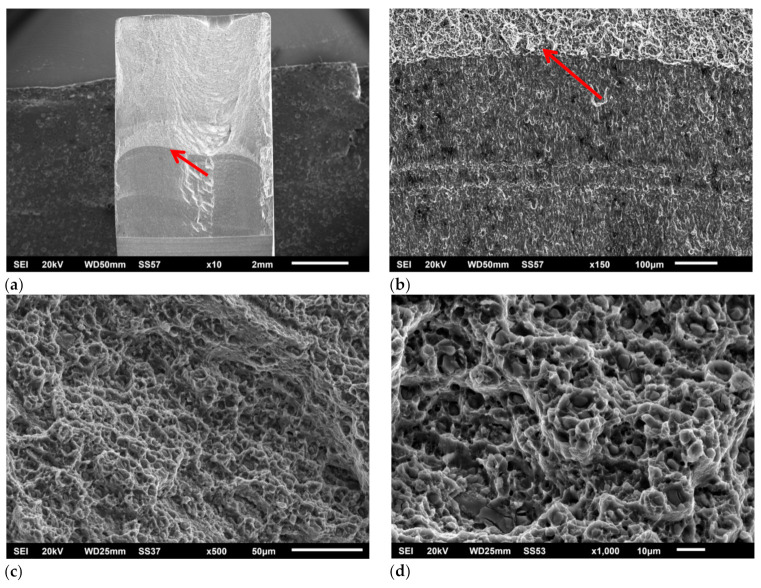
Fracture surface of the B-SZ fracture specimen: (**a**) macro, (**b**) fatigue crack zone and stable crack growth zone, (**c**,**d**) stable crack growth zone.

The fatigue crack propagation surface on the B-AS sample is typical for TMAZ, i.e., a microstructure with inhomogeneous distribution of different size phase particles is shown in Figure 12a. The surface with varying sizes of dimples and fragmented particles in the bottom of the larger dimples is observed. The Figure 12b shows alternately areas with large dimples formed on large secondary phase particles and ridges, consist of elongated, small and shallow dimples formed on precipitates. The fracture surface of the B-SZ specimen is given in Figure 13a, and its fatigue crack zone and stable crack growth zone are given in Figure 13b–d. On the sample B-SZ, a transcrystalline ductile fracture is observed in the fatigue crack propagation zone (red arrow). Dimples contain fragmented particles and precipitates. The fracture surface consists of much shallower and finer dimples (Figure 13c) compared to ones in the TMAZ (Figure 12b). More uniform dimple size can be observed, indicating that the crack propagated through the homogenous SZ (Figure 6c), i.e., the zone with more homogenous size and distribution of the particles. The observed tiny dimples originated/nucleated on precipitates (Figure 13d).

## 4. Conclusions

The research presented in this paper is related to fracture behavior of FSW-AA2024-T351-joints of lightweight structures in the aerospace industry. Friction stir welding is a process of joining materials which results in a welded joint whose mechanical and structural property depend on many mutually conditioned parameters of the welding process. The research was focused on the analysis of the influence of welding speed on fracture behavior of FSW-AA2024-T351. Based on the comprehensive experimental testing and performed fracture analysis related to three different welding speeds (*v* = 73, 116 and 150 mm/min), at constant rotation speed of *n* = 750 rpm the following conclusions can be made:

- Although at the lowest welding speed (the amount of heat generated is the largest) the grain size in the SZ is the largest; the hardness increases as the welding speed decreases, regardless of the coarser grain in the SZ. This tendency suggests that a significant increase in hardness with decreasing welding speed is not a function of grain size, but a function of particle size and distribution of the second phase and precipitate.
- By analyzing the results of fracture toughness, it can be concluded that for all welding samples, the structure of the welded joint on the retreating side has higher fracture toughness, i.e., it has a more pronounced resistance to crack propagation in relation to the welded joint structure on the advancing side.
- If we compare the fracture toughness of structures obtained by different welding parameters at the same position of the welded joint, it can be concluded that the highest fracture toughness has welded joints achieved by welding parameters B-750/116, followed by welded joints obtained by welding parameters A-750/73 and C-750/150, which have approximately 15% lower fracture toughness.

## Figures and Tables

**Table 1:** Chemical composition of AA2024-T351 base materials, data from [42].

Chemical Composition	Al	Cu	Mg	Mn	Fe	Si	Zn	Ti
wt. %	90.7–94.7	3.8–4.9	1.2–1.8	0.3–0.9	0.5	0.5	0.25	0.15

**Table 2:** Mechanical properties of AA2024-T351 base materials, data from [42].

Yield Strength *R_eh_* (Mpa)	Ultimate Tensile Strength *R_m_* (MPa)	Elongation *A*_5_ (%)	Hardness * HB	Hardness HV
≥310	≥425	≥10	120	137

* Typical; 500 g load; 10 mm ball.

**Table 3:** Friction stir welding parameters.

Sample	Tool Rotation Speed *n* rpm	Welding Speed *v* mm/min	Ratio *n*/*v* rev/mm
**A**	750	73	10.27
**B**		116	6.47
**C**		150	5

**Table 4:** Fracture toughness *K_Ic_* of the analyzed FSW-joint for different welding speeds and notch locations.

Sample	*J_Ic_* N/mm	*K_Ic_* N/m^3/2^	*δ_Ic_* mm
A-AS	25	45.3	0.040
A-RS	29	48.8	0.046
B-AS	33.5	52.4	0.043
B-SZ	23	43	0.035
B-RS	39	56.5	0.059
C-AS	24.6	44.9	0.037
C-SZ	23	43.4	0.029
C-RS	28	47.9	0.036
BM	27	47	0.035

## Data Availability

Data sharing is not applicable to this article.
